# P049 Navigating diagnostic uncertainty in paraneoplastic large vessel vasculitis

**DOI:** 10.1093/rap/rkag113.079

**Published:** 2026-11-03

**Authors:** Nishita Padmanabhan, Rachel Brooker, Rosalind Benson

**Affiliations:** NHS University Hospitals of Liverpool Group, Liverpool, United Kingdom; The Clatterbridge Cancer Centre NHS Foundation Trust, Liverpool, United Kingdom; NHS University Hospitals of Liverpool Group, Liverpool, United Kingdom

## Abstract

**Introduction:**

Paraneoplastic large vessel vasculitis (LVV) is a rare but recognised condition. Distinguishing LVV from infection or disease progression related to malignancy can be challenging due to overlapping clinical, biochemical, and imaging features. We present a case of an incidental finding of aortitis in a patient with metastatic olfactory neuroblastoma, where a relapse of her LVV heralded progression of her cancer. This case underscores the difficulties in assessing and managing paraneoplastic LVV and the challenges of treatment in the context of ongoing oncological treatment.

**Case description:**

A female patient with metastatic olfactory neuroblastoma and a history of papillary thyroid carcinoma was referred after the incidental discovery of aortitis on PET-CT during oncological surveillance. During her initial rheumatology assessment, she reported symptoms of fatigue, widespread arthralgia, myalgia, and right hip pain, without any features indicative of cranial giant cell arteritis or other systemic inflammatory diseases. Physical examination revealed no significant findings. A thorough diagnostic workup revealed no infective cause, and a diagnosis of paraneoplastic LVV was made. Oral prednisolone (0.5mg/kg) was started, and she experienced significant clinical improvement, normalisation of inflammatory markers and radiological resolution of the aortic inflammation. Steroids were successfully tapered over 12 months with a successful period of treatment-free remission.

Six months later she reported recurrence of fatigue and loss of appetite. Clinical examination also noted the development of a lesion in the left temporal area. Investigations revealed a rise in inflammatory markers which were significantly higher than at initial presentation. In close discussion with oncology, biopsy of the temporal lesion identified a new metastatic deposit and on repeat PET-CT, aortitis had returned alongside progression of a previously stable humeral metastasis and hypermetabolic changes in the lungs. These pulmonary changes presented a differential diagnosis including inflammation, malignancy and infection. Balancing the risk of immunosuppression and the need to treat active LVV and infection, prednisolone was restarted at 15mg alongside treatment for pneumonia after seeking further advice from the respiratory team. She has since started on a further chemotherapy regimen with the aim of slowing the progression of metastatic neuroblastoma. Low-dose prednisolone has been continued, with normalisation of inflammatory markers and resolution of constitutional symptoms; following chemotherapy, introduction of steroid-sparing agents is planned. Further surveillance PET-CT imaging is planned to assess response to chemotherapy alongside any further evidence of LVV.

**Discussion:**

This case illustrates the complexities of assessing disease activity in LVV within the context of malignancy and the potential for concurrent infection. Olfactory neuroblastoma is typically associated with paraneoplastic endocrinopathies. However, here we describe a case of paraneoplastic LVV demonstrating a distinct pattern of steroid-responsive disease, with relapses occurring following a period of remission in step with progression of her underlying malignancy.

There are limitations to relying on inflammatory markers or imaging alone to evaluate disease activity in LVV, as demonstrated when the clinical picture was complicated by superadded pulmonary infection. The involvement of respiratory and radiology specialists, alongside regular discussions between rheumatology and oncology teams, has underscored the importance of multidisciplinary collaboration in managing complex LVV cases. It is essential to avoid assuming disease relapse solely based on elevated inflammatory markers; careful integration of clinical context and imaging findings is critical before escalating immunosuppressive treatment.

**Key learning points:**

• Paraneoplastic large vessel vasculitis (LVV) should be considered in patients with malignancies presenting steroid-responsive systemic symptoms, particularly when there is a relapse following steroid tapering. The assessment of disease activity remains fundamentally rooted in clinical features and symptom evolution, even in the context of advanced imaging techniques. Elevated inflammatory markers are non-specific and may indicate infection, vasculitis, or both; therefore, they should not be relied upon in isolation to guide treatment decisions.

• Interpreting imaging findings in LVV can be challenging, especially when infection or malignancy is present. Distinguishing between infection and active vasculitis is crucial, as management strategies for each condition are often mutually exclusive. Treatment should be tailored to the individual patient, considering clinical, biochemical, and imaging data over time, rather than relying on assessments from a single time point. In complex cases, a multidisciplinary approach is essential to balance competing clinical risks effectively.

